# Atomic Scissors: A New Method of Tracking the 5-Bromo-2′-Deoxyuridine-Labeled DNA *In Situ*

**DOI:** 10.1371/journal.pone.0052584

**Published:** 2012-12-26

**Authors:** Anna Ligasová, Dmytro Strunin, Radek Liboska, Ivan Rosenberg, Karel Koberna

**Affiliations:** 1 Department of Molecular Cytology and Cytometry, Institute of Biophysics, Academy of Sciences of the Czech Republic, Brno, Czech Republic; 2 Department of RNA Biology, Institute of Molecular Genetics, Academy of Sciences of the Czech Republic, Prague, Czech Republic; 3 Oligonucleotide Group, Institute of Organic Chemistry and Biochemistry, Academy of Sciences of the Czech Republic, Prague, Czech Republic; University Nijmegen Medical Centre, The Netherlands

## Abstract

A new method of the light microscopy detection of BrdU-labeled DNA *in situ* is described. It is based on the oxidative attack at the deoxyribose moiety by copper(I) in the presence of oxygen, which leads to the abstraction of hydrogen atom from deoxyribose culminating in the elimination of the nucleobase, scission of the nucleic-acid strand and formation of frequent gaps. The gaps allow the reaction of the antibodies with the commonly used markers of replication (e.g. 5-bromo-2′-deoxyuridine), which are otherwise masked. The method developed makes it possible to detect nuclear and mitochondrial DNA replication efficiently. In most cases, it does not inhibit effective protein detections and in addition enables simultaneous localization of newly-synthesized RNA. The alternative presently-used methods result in protein denaturation and/or extensive DNA cleavage followed by the DNA-bound proteins peeling off.

## Introduction

5-Bromo-2′-deoxyuridine (BrdU) or its alternatives, 5-chloro-2′-deoxyuridine (CldU) and 2′-deoxy-5-iodouridine (IdU), are widely used for the visualization of sites containing newly replicated DNA. As they are inaccessible in double-stranded DNA for a reaction with an antibody, it is necessary to use special steps to make them accessible [1]–[5]. The examples include the use of hydrochloric-acid solutions resulting in depurination and cleavage of the DNA [2]–[4], the use of sodium hydroxide resulting in a loosening of the DNA structure as a consequence of the deprotonation of the nucleobases [1], [4], [5] or the use of DNase I or a mixture of nucleases [2], [4]. The disadvantage of the systems based on the use of mineral acids and hydroxides is the fact that both agents cause considerable changes in the cell structure accompanied by strong and uncontrolled destruction not only of DNA and RNA but also of proteins [6]. The use of DNase I or the mixture of nucleases has another disadvantage as it usually provides only a weak signal when compared to the signal obtained using acids or hydroxide. Moreover, we have shown here that it results in an extensive peel-off of at least some DNA-bound proteins.

Although a technique based on the reaction of 2′-deoxy-5-ethynyluridine (EdU) that bypasses the necessity to use antibodies owing to the possibility of the reaction of the terminal alkyne of EdU with the azido group attached to the fluorescent dye has been developed [7], it suffers from the fact that EdU can exhibit cytotoxicity with an unknown mechanism that varies for different cell lines [8], [9]. Moreover, it seems that the sensitivity of the microscopic detection of the mitochondrial replication by EdU is lower than by BrdU. Unlike BrdU, EdU obligatorily requires an amplification step (compare e.g. [10]–[12]). Additional methods, like the use of biotinylated nucleotides, require specific steps for their introduction and do not make it possible to control the time of their incorporation [13], [14]. Finally, the BrdU is relatively cheap as compared to EdU or labeled nucleotides. We have developed a completely new approach for tracking the BrdU-labeled DNA in cells based on the scission of DNA by a radical-oxidative reaction on the carbon atoms of the deoxyribose followed by a set of elimination reactions including the elimination of the nucleobases, finally resulting in the cleavage of the internucleotide linkages [15] and formation of gaps. The elaborated system is based on copper(I) ions and oxygen. Although the system of oxidative attack at the deoxyribose moiety by copper or iron ions, sodium ascorbate and hydrogen peroxide for isolated DNA as an effective DNA cleavage system was described earlier [16], and the gaps created by the oxidative attack at the deoxyribose moiety can theoretically make incorporated 5-halo-nucleosides accessible, it was not clear how effective the formation of gaps in DNA is in fixed cells, what is the sensitivity of this approach, and what the effect of the conditions used is on the cellular structure.

Presently, the mechanism of gap formation is not completely resolved. It is supposed that a Fenton reaction is employed during the DNA cleavage when hydroxyl radicals are created in the presence of copper or iron ions and hydrogen peroxide [17]–[19]. Likewise, some data indicate the possibility of the participation of cupryl ions (Cu(III)aq, [20]) or a complex of copper and oxygen in this reaction (copper-oxo complex, [21], [22], [23]). The system based on copper ions was chosen in this study as the experiments performed so far have shown that the system based on copper ions, sodium ascorbate and hydrogen peroxide is more efficient than the system based on iron ions [24]. For validation of the method developed, we have compared it with the methods presently used for the detection of replicated DNA by means of 5-halo-nucleosides.

## Materials and Methods

### Cell culture, DNA and RNA labeling, fixation and permeabilization protocol

Human HeLa cells (a generous gift from Dr. David Staněk, Institute of Molecular Genetics, Prague) were cultured on coverslips in a Petri dish in Dulbecco's modified Eagle's medium with L-glutamine (DMEM, Gibco) supplemented with 10% fetal calf serum (PAA Laboratories), 1% gentamicin and 0.85 g/l of NaHCO_3_ at 37°C in a humidified atmosphere containing 5% CO_2_.

For the labeling of DNA replication, BrdU or CldU or IdU were added to the culture medium for 10 minutes (if not stated otherwise). The final concentration of all 5-halo-nucleosides was 20 µM. For the labeling of newly synthesized RNA, 5-ethynyluridine (EU) was added to the culture medium for 20 minutes. The final concentration of EU was 0.5 mM. In the experiments focused on the detection of the replication of the mitochondrial genome, BrdU was added to the cell in medium for 1 or12 hours (the final concentration was 20 µM).

The cells were fixed with 2% formaldehyde for 10 minutes, washed in 1× PBS, permeabilized in 0.2% Triton X-100 for 10 minutes and washed in 1× PBS.

### Cleavage of cellular DNA by copper(I) ions

#### A. Testing the optimal cleavage procedure

The fixed and permeabilized cells were briefly washed in 1× PBS and then three times in distilled water. Subsequently, the cells on coverslips were incubated in the cleavage solution in a Petri dish for 30 minutes (if not stated otherwise) at room temperature (RT) on the laboratory shaker. The following cleavage solutions were tested for the optimal DNA-cleavage procedure:

- distilled water, 10 mM sodium ascorbate, alternatively 0.25 mM, 2 mM, 4 mM, 6 mM copper(II) sulfate and alternatively 0.05%, 0.1%, 0.5%, 1%, 2% and 3% hydrogen peroxide
- distilled water, 10 mM sodium ascorbate and alternatively 0.25 mM, 2 mM, 4 mM, 6 mM, 8 mM, 10 mM and 20 mM of copper(II) sulfate;
- distilled water, 200 mM Hepes, 10 mM sodium ascorbate and 4 mM copper(II) sulfate;
- distilled water, 1 mM Tris, 10 mM sodium ascorbate and 4 mM copper(II) sulfate;
- alternatively 2 mM, 4 mM, 6 mM, 8 mM, 10 mM and 20 mM of tetrakis(acetonitrile)copper(I) hexafluorophosphate (Sigma Aldrich) in distilled water.

According to our results, the copper(I) ions obtained by the reduction of copper(II) sulfate by means of sodium ascorbate or by the dissolution of tetrakis(acetonitrile)copper(I) hexafluorophosphate provided similar results.

In experiments focused on the effect of the reduction of the DNA-bound copper(II) ions, the cells were first incubated in 4 mM copper(II) sulfate for five minutes, washed three times in distilled water and then incubated in 10 mM sodium ascorbate for five or ten or thirty minutes.

#### B. Standard cleavage procedure

According to the results obtained, we have formulated the standard cleavage procedure.

One-step procedure: The fixed and permeabilized cells were briefly washed three times in distilled water and subsequently incubated in a cleavage solution consisting of 10 mM sodium ascorbate and 4 mM copper(II) sulfate (30 minutes, RT) on the laboratory shaker. After that, the cells were washed in 20 mM EDTA (30 minutes, RT) on the shaker. For the detection of mitochondrial DNA, only 60 seconds of incubation in the cleavage solution was used.

Two-step procedure: In the two-step procedure, an exonuclease treatment was used to enhance the signal. In this case, the cells were incubated for ten minutes in the above-described cleavage solution on the shaker followed by washing in 20 mM EDTA (30 minutes, RT) on the shaker and then by a 30-minute incubation with a mixture of antibody and exonuclease III (1 U/µl, Fermentas) and 1× buffer for exonuclease III at RT. For the detection of mitochondrial replication, only 60 seconds of incubation in the cleavage solution was used.

### Cleavage of plasmid DNA

The plasmid DNA pEXP5-NT/CALM3, 3195 bps long, (Invitrogen) was linearized by EcoRI (FastDigest Enzymes, Fermentas) prior to use and dissolved in water to a concentration of 150 ng/µl. 300 ng of DNA were added to each reaction. The cleavage reaction was performed in a cleavage solution consisting of 1 mM sodium ascorbate and 0.4 mM copper(II) sulfate. The reaction was stopped by adding 25 mM EGTA. The samples were dialyzed on the dialysis membrane (0.025 µm VSWP MF-membrane filters, MILLIPORE) against 0.1 M Tris-HCl for 25 minutes. The dialyzed DNA was put directly to the 1% agarose gel with a voltage of 5 V/cm. The gel was stained by means of ethidium bromide, and the images were taken under UV light.

### The role of the superoxide anion, hydroxyl radical and singlet oxygen in DNA cleavage and the creation of abasic sites

**Figure 1:**
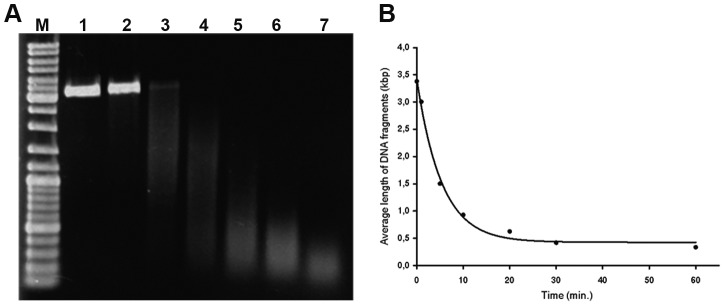
The cleavage of plasmid DNA using copper(II) sulfate and sodium ascorbate in presence of oxygen. **A**) The results of the cleavage of the plasmid DNA are shown. The plasmid DNA (pEXP5-NT/CALM3) was incubated in an aqueous solution of copper(II) sulfate and sodium ascorbate in the presence of oxygen for 0, 1, 5, 10, 20, 30 and 60 minutes (lines 1, 2, 3, 4, 5, 6 and 7, respectively). Line M represents the DNA molecular mass marker (GeneRuler™ DNA ladder Mix, Fermentas). The lowering of integral intensity toward 60 minutes apparently resulted from the increase of the low-weight DNA that escaped the gel. **B**) The centers of density of the bands from the above-mentioned experiments (**a**) plotted against the time of the incubation are shown.

If the effect of oxygen radicals was studied, the following reaction mixtures were used:

1. 1 mM sodium ascorbate, 0.4 mM copper(II) sulfate and 1 µg/ml SOD (Sigma Aldrich);
2. 1 mM sodium ascorbate, 0.4 mM copper(II) sulfate and 200 mM Hepes;
3. 1 mM sodium ascorbate, 0.4 mM copper(II) sulfate and 100 mM Tris-HCl, pH 7.4;
4. 1 mM sodium ascorbate, 0.4 mM copper(II) sulfate and alternatively 1 mM and 10 mM DMSO;

In the experiments concerning the abasic site, the samples were incubated first in 1 mM sodium ascorbate, 0.4 mM copper(II) sulfate solution for 30 minutes and then with 1 M piperidine for 30 minutes at 90°C [25]. The centers of density of the bands in the graph in Figure 1B were evaluated from the gel electrophoresis image by means of ImageJ software. The centers of density have been found for each track, plotted against the time of incubation and fitted to the exponential decay regression described by the quotation: 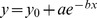, where *y* is equal to the average length of DNA fragments and *x* to the incubation time. Parameter *a* represents the amplitude, parameter *b* the degradation halftime and parameter y_0_ the background correction.

### Enzymes used

These enzymes and condition were used:

1. Terminal deoxynucleotidyl transferase (TdT; 2 U/µl, 10 minutes, 37°C, Fermentas), buffer for TdT, 0.05 mM dATP, dGTP, dCTP and 0.05 mM Alexa Fluor® 555-aha-2′-deoxyuridine-5′-triphosphate (Alexa-dUTP);
2. DNA polymerase I (0.2 U/µl, 10 minutes, RT, Fermentas), buffer for DNA polymerase I, 0.05 mM dATP, dGTP, dCTP and 0.05 mM Alexa-dUTP;
3. Klenow fragment (0.2 U/µl, 10 minutes, RT, Fermentas), buffer for the Klenow fragment, 0.05 mM dATP, dGTP, dCTP and 0.05 mM Alexa-dUTP;
4. Klenow fragment Exo- (0.2 U/µl, 10 minutes, RT, Fermentas), buffer for the Klenow fragment Exo-, 0.05 mM dATP, dGTP, dCTP and 0.05 mM Alexa-dUTP;
5. Exonuclease III (1 U/µl, 30 minutes, RT, Fermentas), buffer for exonuclease III;
6. Exonuclease λ (0.1 U/µl, 30 minutes, RT, Fermentas), buffer for exonuclease λ;
7. Shrimp alkaline phosphomonoesterase (phosphatase; SAP; 1 U/µl, 20 minutes, 37°C, Fermentas), buffer for SAP.

### Antibodies

These primary antibodies were used:

Rat anti-BrdU antibody (1∶100, used in most of experiments, Abcam), mouse anti-BrdU antibody (1∶200, co-localization of BrdU-labeled DNA and EU-labeled RNA, Exbio), chicken anti-BrdU antibody (1∶100, detection of BrdU-labeled DNA in mitochondria, Abcam), mouse anti-SC35 antibody (1∶250, Abcam), rabbit anti-H1.2 antibody (1∶100, Abcam), mouse anti-PCNA antibody (proliferating cell nuclear antigen; 1∶100, Abcam), rabbit anti-Cdc45 antibody (1∶100, Santa Cruz Biotechnology), mouse anti-coilin antibody (1∶50, Abcam) and mouse anti-mitochondrial antibody (MTC02 antibody; 1∶20, Abcam).

These secondary antibodies were used:

Cy3 anti-mouse, DyLight 649 anti-mouse, Alexa Fluor® 488 anti-mouse, Cy3 anti-rabbit, Cy3 anti-rat, FITC anti-rat, DyLight 649 anti-rat, DyLight 649 anti-chicken and FITC anti-rabbit antibodies (all: 1∶100, Jackson ImmunoResearch).

All antibodies were diluted in 1× PBS if not stated otherwise. The cells were incubated with primary and secondary antibodies for 30 minutes at RT if not stated otherwise. After washing, the cells were mounted in Mowiol and inspected by means of fluorescence microscopy.

### Alternative procedures

For the detection of BrdU or CldU or IdU, the fixed cells were prior to incubation with the antibody incubated in the following solutions:

1. 4 N (nuclear replication) or 0.5 M (mitochondrial replication, [12]) HCl, 20 minutes, RT;
2. 0.07 M NaOH, 3 minutes, RT;
3. 20 U/ml DNase I (Sigma Aldrich), 30 minutes, 37°C [26]. In this case, the primary antibody was alternatively added directly to the DNase I. Both protocols provided similar signal intensity.

We tested several concentrations of HCl, NaOH and DNase I. The concentrations used represented the optimum with respect to the signal∶noise ratio. Alternatively, a labeling kit based on non-specified nuclease activities from Roche was used according to the suppliers' protocol, but it required ethanol fixation and no signal was observed in formaldehyde-fixed cells.

The nuclear DNA was stained by DAPI (0.3 µM, 20 minutes, RT).

### Detection of newly-synthesized transcripts and polyadenylated RNA

The incorporated EU was detected by means of a click reaction with the Alexa Fluor® 488 azide using the commercially-available kit according to the suppliers' protocol (30 minutes, RT, Invitrogen). The click reaction preceded the treatments with copper(I) or 4 N HCl or incubation with DNase I. In the case of control cells no treatment was performed. In the case of polyadenylated RNA, fixed and permeabilized cells were treated with 4 N HCl, copper(I) ions or were untreated (control). The reverse transcription was performed according to [27].

After washing, the cells were mounted in Mowiol and inspected by means of fluorescence microscopy.

### Image acquisition and processing

The images were obtained by means of an Olympus IX81 microscope (Olympus) equipped with a Hamamatsu ORCA II camera with a resolution of 1344×1024 pixels using Cell∧R acquisition software. The following objectives were used: UPLFLN oil 40× NA 1.3 and UPLSAPO oil 100× NA 1.4.

For image processing, Adobe Photoshop software was used. For drawing the models, Rhinoceros software was used. For drawing the graphs, Microsoft Excel and Sigma Plot software were used.

The signal intensities in the regions of interest (ROI) were measured using the Cell∧R acquisition software. These kinds of ROIs were evaluated: the nucleoplasm for the evaluation of the replication signal (acquisition time: 99 ms), the signals provided by anti-PCNA antibody (acquisition time: 83 ms) and anti-Cdc45 antibody (acquisition time: 170 ms), the speckles for the evaluation of signal provided by anti-SC35 antibody (acquisition time: 7 ms), the spots corresponding to the coiled bodies for the evaluation of the signal provided by anti-coilin antibody (acquisition time: 4 ms), the nuclei for the evaluation of the signal provided by DAPI staining (acquisition time: 9 ms), the signal provided by anti-H1.2 antibody (acquisition time: 20 ms) and the signal provided by incorporated EU (acquisition time: 17 ms). Twenty images were acquired from every sample and twenty nuclei were evaluated in every such image for the determination of the mean signal intensity (400 nuclei in total).

## Results

### Effective DNA cleavage occurs only in the presence of oxygen

We have used two systems to test the efficiency of the DNA cleavage by copper(I) ions. The first was based on the isolated DNA and the size analysis of the products; the second was based on the enzymatic detection of DNA gaps in the formaldehyde-fixed and permeabilized cells by means of DNA polymerase I mediated incorporation of fluorescently labeled nucleotides. As we found that the addition of even less than 0.1% hydrogen peroxide had a very destructive impact on the detection of proteins, the DNA cleavage was performed without the addition of hydrogen peroxide. Our results have shown that already less than one milimolar concentration of copper(I) ions leads to extensive cleavage of isolated DNA (Figure 1). The mixture used contains as its standard 1 mM sodium ascorbate and 0.4 mM copper(II) sulfate for the cleavage of isolated DNA. *In situ* experiments were conducted with concentrations that were ten times higher. We tested lower and higher concentrations of copper(I) ions for *in situ* experiments as well. However, the lower concentrations led to a progressive decrease of the signal. The higher concentrations did not result in a signal increase.

According to our results the prolongation of the incubation time of isolated DNA in the solution containing copper(I) ions results in a gradual increase of DNA cleavage (Figure 1A, B). Similar time dependence has been observed in the experiments with fixed cells. Quantification of the experiments with fixed cells was performed by means of the measurement of the fluorescence signal provided by DNA polymerase I. According to our results the 30-minute incubation in the cleavage solution provided approximately 2.4±0.43 times higher signal than the 10-minute incubation of samples in the cleavage solution.

For a successful *in situ* detection of DNA gaps, the presence of oxygen was necessary. When the influence of oxygen was studied, the samples were incubated on the laboratory shaker or the cleavage solutions were bubbled by gaseous nitrogen for five minutes before and also during the incubation of the samples in the cleavage solution. According to our results, the incubation on the laboratory shaker was sufficient for an efficient oxygen supply and the subsequent DNA polymerase I labeling. In the samples where the cleaving mixture was bubbled during the incubation by gaseous nitrogen, no signal was observed. In that case, we obtained the signal only when the samples were further incubated with the oxidation agent, Oxone (potassium monopersulfate, Sigma Aldrich). In this experiment, the cells were briefly rinsed in distilled water after incubation in the cleavage solution, then incubated for 30 minutes in the 4 mM solution of Oxone and finally briefly washed three times in distilled water.

We found that the prolongation of the sample incubation in the solution containing copper ions resulted in an even up to brownish coloration of the permeabilized cells. This coloration was removed by washing the samples alternatively in a solution containing 20 mM EDTA or 20 mM EGTA on the shaker for 30 minutes. We did not study whether these products contain metal, monovalent or bivalent copper. However, the necessity of the presence of oxygen for the successful removal of these products more likely denoted the presence of reduced forms of copper ions. The presence of these copper products inhibits the reaction with antibodies against selected nuclear proteins. However, after the removal of the copper products, the reaction was restored to an extent in copper(I) untreated cell samples.

**Figure 2:**
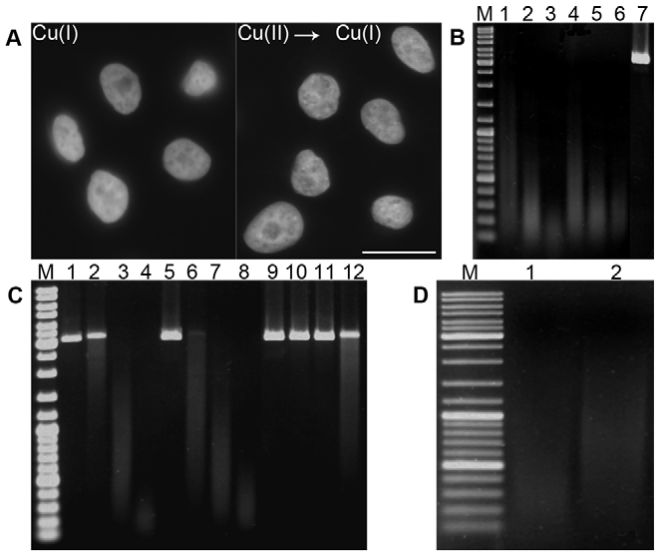
The gaps detection, the effect of SOD, Hepes and Tris-HCl and the abasic sites detection. **A**) The detection of the gaps produced by the incubation of cells with 4 mM copper(I) for 30 minutes or by the incubation of cells in the solution of 4 mM copper(II) sulfate for 5 minutes, followed by the reduction of the DNA-bound bivalent copper ions by 10 mM sodium ascorbate for 5 minutes is shown. The gaps were visualized by means of DNA polymerase I and Alexa-dUTP. Bar: 20 µm. **B**) The effect of SOD on plasmid DNA cleavage is shown. The plasmid DNA was incubated in the cleavage solution for 5, 10 and 30 minutes without (lines 1, 2, 3, respectively) or with SOD (lines 4, 5, 6, respectively). Line 7 represents the copper(I) untreated sample; line M represents the DNA molecular mass marker. Only a very low inhibition of DNA cleavage by SOD was observed. **C**) The effect of Hepes and Tris-HCl is shown. The plasmid DNA was incubated in cleavage solution for 0, 5, 10 and 30 minutes alone (lines 1, 2, 3, 4, respectively), with 0.2 M Hepes (lines 5, 6, 7, 8, respectively) or with 0.1 M Tris-HCl (lines 9, 10, 11, 12, respectively). Line M represents the DNA molecular mass marker. Only Tris-HCl efficiently blocked DNA cleavage. **D**) The effect of piperidine is shown. The plasmid DNA was incubated in cleavage solution for 30 minutes and subsequently in 1 M piperidine (line 1) or distilled water (line 2) at 90°C. Line M represents the DNA molecular mass marker. The shift to the shorter DNA fragments after piperidine treatment indicates formation of abasic sites.

We have further found that the samples can be first incubated in a solution of bivalent copper ions. Subsequently, the copper(II) ions bound to DNA are reduced to monovalent ions and cleavage is induced by means of incubation in an oxygen-containing environment (Figure 2A).

### The role of the superoxide anion, hydroxyl radical and singlet oxygen in DNA cleavage and the creation of abasic sites

To evaluate the influence of the superoxide anion, plasmid DNA was incubated in the cleavage solution supplemented with superoxide dismutase (SOD), an enzyme that changes the superoxide anion to oxygen and hydrogen peroxide. The experiment showed a very low inhibition of DNA cleavage (Figure 2B). It indicates that, although the superoxide anion plays a role in this reaction, it is not the primary ion responsible for the cleavage. When we utilized the commonly used scavenger of hydroxyl radicals, DMSO, no effect on the DNA cleavage was observed. An addition of the scavenger of singlet oxygen, Tris-HCl, to the cleavage solution resulted in a clear reduction of DNA cleavage (Figure 2C), strongly indicating the important role of singlet oxygen in DNA cleavage.

To find whether abasic sites are created during DNA cleavage, we incubated plasmid DNA first in the standard cleavage solution and subsequently also with 1 M piperidine (Figure 2D). As such a treatment cleaves DNA at abasic sites [25] and our results showed an increased cleavage of treated DNA as compared to untreated DNA (Figure 2D), it is obvious that during copper(I)-mediated DNA cleavage also abasic sites are formed.

### The DNA gaps contain phosphate groups at the 3′ ends

**Figure 3:**
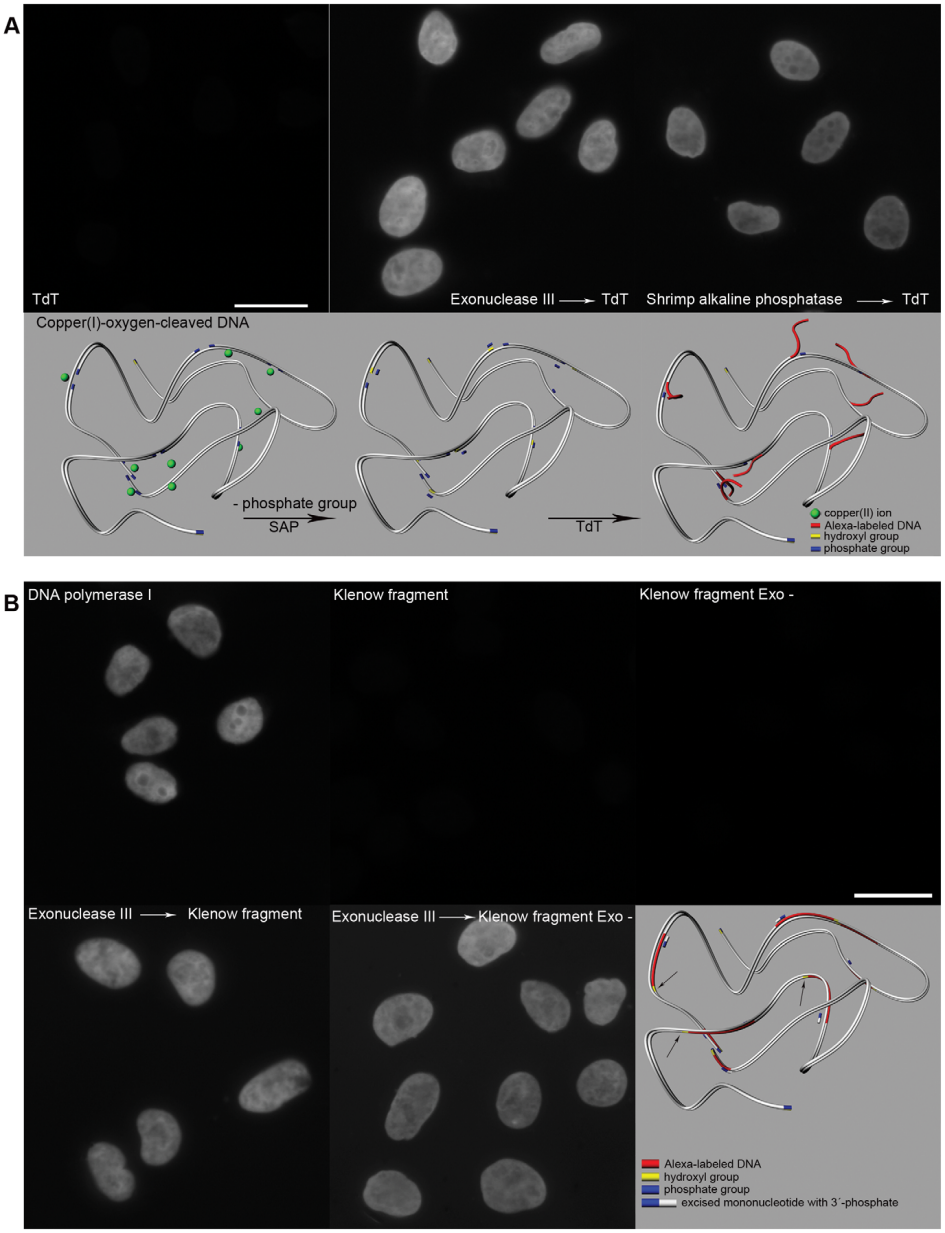
Copper(I) treatment produces short gaps with phosphate groups at the 3′ end. **A**) TdT was used to incorporate Alexa-dUTP at the 3′ end of the gaps. A strong signal is observed only after the pre-incubation of cells with exonuclease III or SAP. The model shows the situation after the action of SAP in the case of double-stranded DNA with several gaps. Although the phosphate groups are shown also at the 5′ end of the gaps, it is not clear whether they are present there. Therefore, the action of SAP is shown for 3′ phosphate groups exclusively. Bar: 20 µm. **B**) DNA polymerase I, Klenow fragment and Klenow fragment Exo- were used to incorporate Alexa-dUTP at the gap sites produced by monovalent copper. Only DNA polymerase I produced a strong signal. When incubation with exonuclease III preceded the polymerase step, a strong signal was observed also in the case of both Klenow fragments. The model shows the action of DNA polymerase I at the sites of created gaps. Both 3′-5′ proofreading activity enabling hydroxyl group formation and 5′-3′ exonuclease activity (for the sake of simplicity, the excised nucleotides are not shown in the model) enabling nick translation are necessary. As no ligase activity was present, nicks at the ends of the labeled chains persisted (arrows in the model picture), although it is not apparent. Bar: 20 µm.

We incubated HeLa cells in the standard cleavage solution and subsequently with TdT and a mixture of deoxynucleotide triphosphates with Alexa-dUTP (Figure 3A). We observed a signal exclusively when the incubation with TdT followed the incubation with exonuclease III (3′-5′ exonuclease; Figure 3A) or SAP (Figure 3A). It clearly testifies to the presence of phosphate groups at the 3′ ends of the gaps created.

### The majority of the gaps are represented by short gaps

When DNA polymerase I was used for DNA labeling; we obtained a very strong signal (Figure 3B). When the Klenow fragment, lacking 5′-3′ exonuclease activity, or the Klenow fragment Exo-, lacking both 5′-3′ exonuclease and 3′-5′ proofreading activity, was used, we did not observe any signal (Figure 3B). The result with the Klenow fragment Exo- because of its inability to restore the hydroxyl group at the 3′ end was in line with the results obtained when using TdT. These results also showed that 5′-3′ exonuclease activity is necessary for the effective DNA labeling. This strongly indicates that the majority of the gaps are in the form of short gaps. Next, we incubated the cells first with the copper(I) ions, then with exonuclease III and subsequently with Klenow fragment or Klenow fragment Exo- (Figure 3B). We observed a strong signal in the experiments with both types of Klenow fragment (Figure 3B), which clearly demonstrates that the majority of the gaps are in the form of relatively short gaps.

### The presence of numerous gaps in DNA enables the detection of 5-halo-nucleosides incorporated in DNA

**Figure 4:**
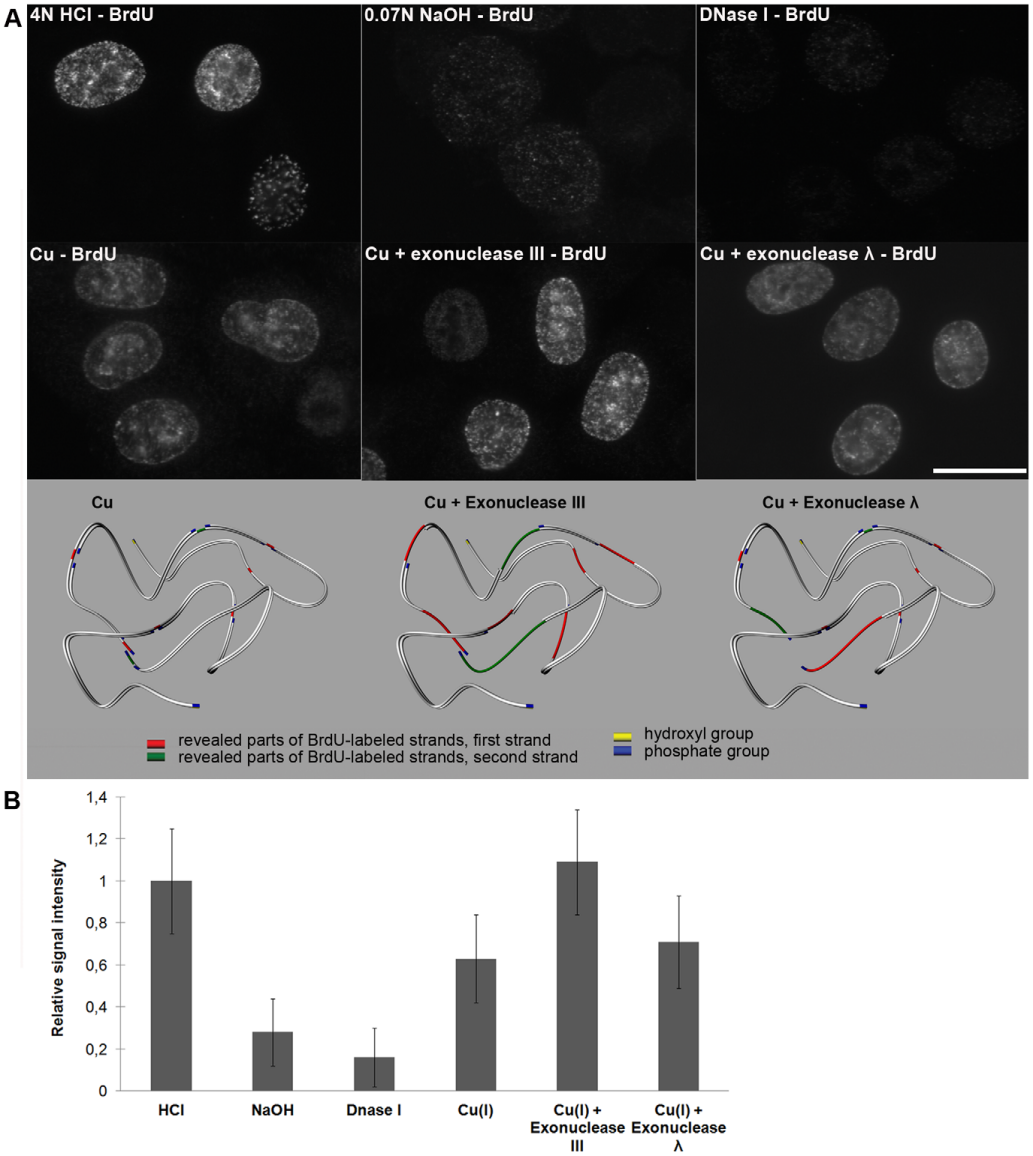
Copper(I)-oxygen efficiently reveals incorporated BrdU; the revelation can be further increased by means of exonucleases. **A**) The results of the detection of the BrdU labeling of replicated DNA using acid (4 N HCl) or hydroxide (0.07 M NaOH) or DNase I treatment or the one-step or the two-step procedure are shown. All of the images were taken using 99-ms time to be able to compare the signal intensity. In the one-step procedure (the image labeled as Cu), the 30-minute treatment with copper(I)-oxygen was used exclusively. In the two-step protocol, a 10-minute treatment of the samples with copper(I)-oxygen was followed by incubation with exonuclease III or exonuclease λ. The model shows the situation for both one-step and two-step procedures. Note that exonuclease λ reveals BrdU-labeled parts in the proximity of close single gaps as it has no activity at nicks and limited activity at gaps. Only close single gaps can result into the formation of double-strand break. Although only one strand is usually labeled by BrdU, the situation is shown as if both strands were labeled in the schematic picture. The revealed parts of distinct strands are distinguished by colors. Bar: 20 µm. **B**) Relative signal intensity is shown in the graph.

We labeled HeLa cells with BrdU, fixed and incubated them with various concentrations of copper(II) sulfate and sodium ascorbate in the presence or absence of oxygen. After BrdU immunodetection, we observed a signal that depended on the incubation time and the concentration of copper ions only in the presence of oxygen. Using a 4–10 mM concentration of copper ions, a sufficient incubation time was around 30 minutes. In the control experiments, we made BrdU accessible for antibodies by means of 4 N HCl, 0.07 M NaOH or 20 U/ml DNase I (Figure 4A). It is obvious that the approach employing copper(I) ions enables a similarly effective detection of incorporated BrdU like 4 N HCl and a much more effective detection than the NaOH and DNase I treatment (Figure 4 A,B). According to the results, only the treatment with NaOH resulted in a significant cell loss during treatment and progressive swelling of the nuclei. We also found that the incubation with exonucleases enables a shortening of the incubation time with copper(I) ions to 5–10 minutes, no matter if exonuclease III or exonuclease λ is used (Figure 4A). The experiments with exonuclease λ, which is inactive on nicks and exhibits only very limited activity on the gaps, indicated that, besides the presence of one-strand gaps, the incubation of cells with monovalent copper ions leads to the formation of frequent double-stranded breaks, probably as a consequence of the separation of DNA strands between close single gaps at opposite DNA strands. It can also explain the lower signal after the use of exonuclease λ compared to the use of exonuclease III.

Further, we compared a minimal incorporation time necessary for the detection of the BrdU signal using copper(I)-mediated cleavage and HCl. We found that this time varies between three and four minutes in both cases of BrdU revelation.

Similar results were obtained also with CldU- and IdU-labeled cells (not shown).

### A comparison of the method developed with those currently used

It was evident from our previous results that copper(I)-based method of the visualization of replication provides strong signals. However, it was not clear how it influences the detection of various cellular proteins and what its sensitivity is as compared to the methods of replication detection used now.

**Figure 5:**
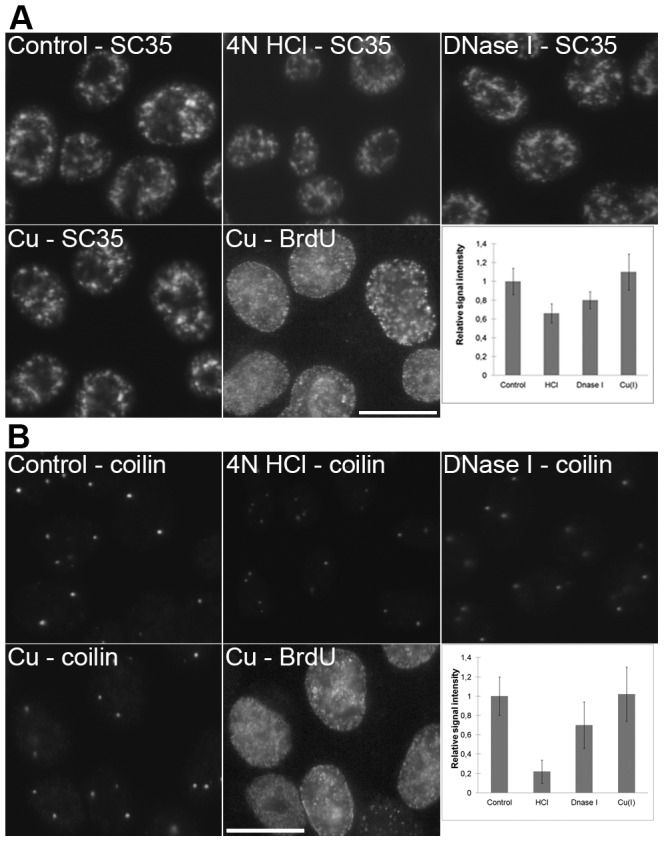
A comparison of the methods based on HCl, DNase I and copper(I) ions. **A**) The detection of SC35 protein in non-treated cells (control; the cells were just fixed and permeabilized without any additional treatment), cells treated with 4 N HCl, DNase I or with a standard two-step procedure (Cu – SC35) is shown. All of the images have been acquired at 7 ms. A simultaneous detection of the BrdU-labeled newly replicated DNA in copper-treated cells from the Cu – SC35 image is shown in the image labeled as Cu – BrdU (the acquisition time was 99 ms). The graph shows the relative signal intensities of particular kinds of the treatments used. Bars: 20 µm. **B**) The detection of coilin protein in non-treated cells (control; the cells were just fixed and permeabilized without any additional treatment), cells treated with 4 N HCl, DNase I or with a standard two-step procedure (Cu – coilin) is shown. All of the images have been acquired at 4 ms. A simultaneous detection of the BrdU-labeled newly replicated DNA in copper-treated cells from the Cu – coilin image is shown in the image labeled as Cu – BrdU (the acquisition time was 99 ms). The graph shows the relative signal intensities of particular kinds of the treatments used. Bars: 20 µm.

**Figure 6:**
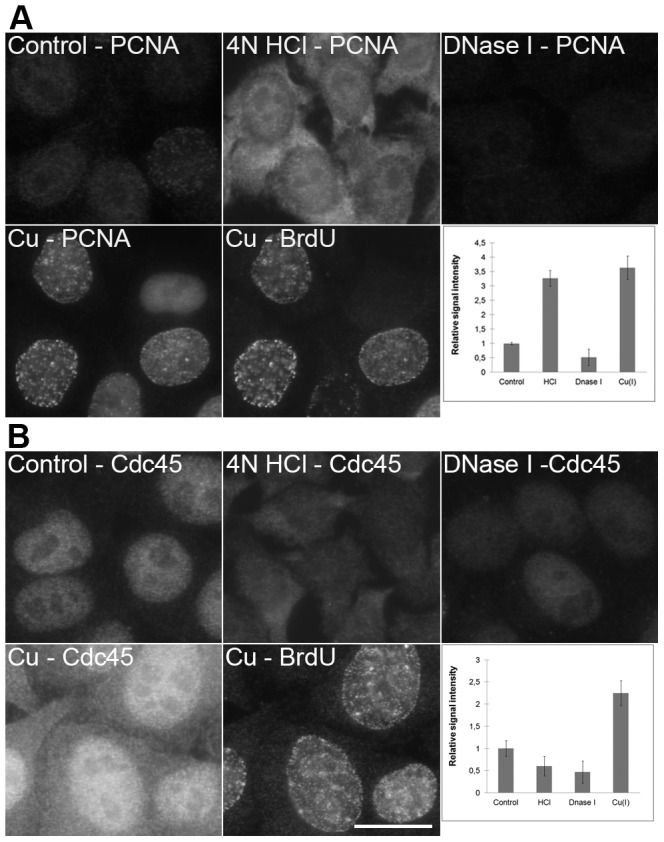
A comparison of the methods based on HCl, DNase I and copper(I) ions. **A**) The detection of PCNA in non-treated cells (control; the cells were just fixed and permeabilized without any additional treatment), cells treated with 4 N HCl, DNase I or with a standard two-step procedure (Cu – PCNA) is shown. All of the images have been acquired at 83 ms. A simultaneous detection of the BrdU-labeled newly replicated DNA in copper-treated cells from the Cu – PCNA image is shown in the image labeled as Cu – BrdU (the acquisition time was 99 ms). The graph shows the relative signal intensities of particular kinds of the treatments used. Bars: 20 µm. **B**) The detection of Cdc45 protein in non-treated cells (control; the cells were just fixed and permeabilized without any additional treatment), cells treated with 4 N HCl, DNase I or with a standard two-step procedure (Cu – Cdc45) is shown. All of the images have been acquired at 170 ms. A simultaneous detection of the BrdU-labeled newly replicated DNA in copper-treated cells from the Cu – Cdc45 image is shown in the image labeled as Cu – BrdU (the acquisition time was 99 ms). The graph shows the relative signal intensities of particular kinds of the treatments used. Bars: 20 µm.

**Figure 7:**
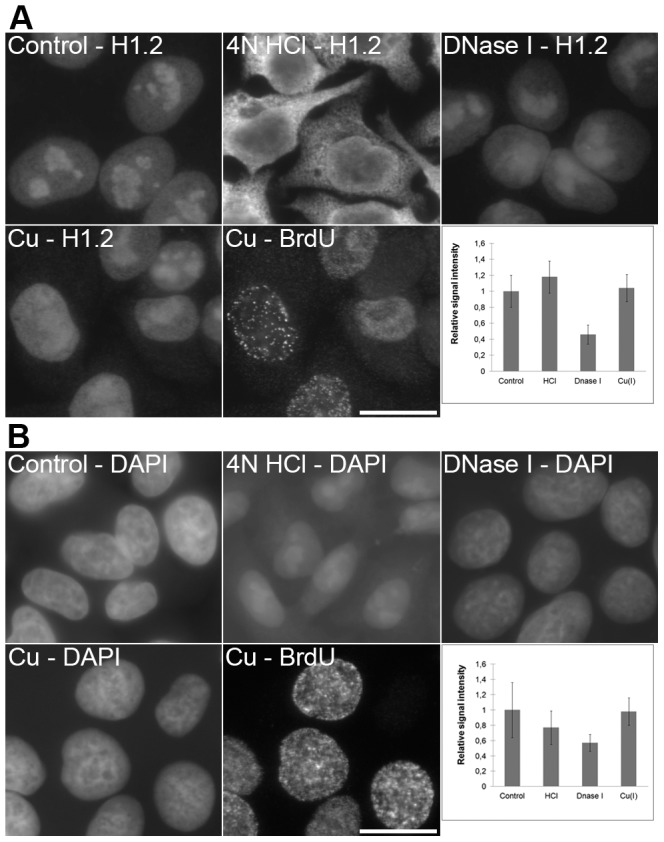
A comparison of the methods based on HCl, DNase I and copper(I) ions. **A**) The detection of H1.2 protein in non-treated cells (control; the cells were just fixed and permeabilized without any additional treatment), cells treated with 4 N HCl, DNase I or with a standard two-step procedure (Cu – H1.2) is shown. All of the images have been acquired at 20 ms. A simultaneous detection of the BrdU-labeled newly replicated DNA in copper-treated cells from the Cu – H1.2 image is shown in the image labeled as Cu – BrdU (the acquisition time was 99 ms). The graph shows the relative signal intensities of particular kinds of the treatments used. Bars: 20 µm. **B**) The detection of DNA in non-treated cells (control; the cells were just fixed and permeabilized without any additional treatment), cells treated with 4 N HCl, DNase I or with a standard two-step procedure (Cu – DAPI) is shown. All of the images have been acquired at 9 ms. A simultaneous detection of the BrdU-labeled newly replicated DNA in copper-treated cells from the Cu – DAPI image is shown in the image labeled as Cu – BrdU (the acquisition time was 99 ms). The graph shows the relative signal intensities of particular kinds of the treatments used. Bars: 20 µm.

We have performed a set of protein-localization experiments under conditions typical for the methods based on the copper(I)-mediated cleavage, DNase I cleavage and HCl treatment (Figures 5, 6, 7). We have measured the mean intensity in the regions typical for the localization of the proteins tested (see Material and Methods). We have found that the mean signal intensity highly depends on the protein target. In the case of the splicing factor SC35 (Figure 5A) and coilin – a marker of coiled bodies (Figure 5B), the decrease of the signal has been observed only in the case of the use of HCl and DNase I. This decrease is higher for HCl. No such decrease has been observed in samples treated with copper(I).

Surprising data were received from the experiments with PCNA (Figure 6A) and Cdc45 protein (Figure 6B). The conditions used during copper(I) treatment led to an increase in the effectiveness of the detection of both proteins The most intensive PCNA signal was observed in domains exhibiting replication activity (Figure 6A). The acid treatment resulted in a complete loss of the specific nuclear signals of both proteins and extensive labeling of cytoplasm. DNase I treatment resulted in a substantial decrease of both PCNA and Cdc45 signals. When histone H1.2 was detected (Figure 7A), the copper(I) treatment did not change the signal intensity while the acid treatment led to a complete loss of the specific signal and an increase of the signal in the cytoplasm. The DNase I treatment resulted in the signal reduction especially outside of the nucleolus. As the significant population of these proteins is bound to DNA, the differences in their localization could be attributed to the differences in the loss of DNA during various procedures. Indeed, DAPI staining revealed progressive changes in the labeling pattern of nuclear DNA after acid treatment and a significant loss of DNA after DNase I treatment (Figure 7B). No loss of DNA was observed after copper(I) treatment (Figure 7B).

**Figure 8:**
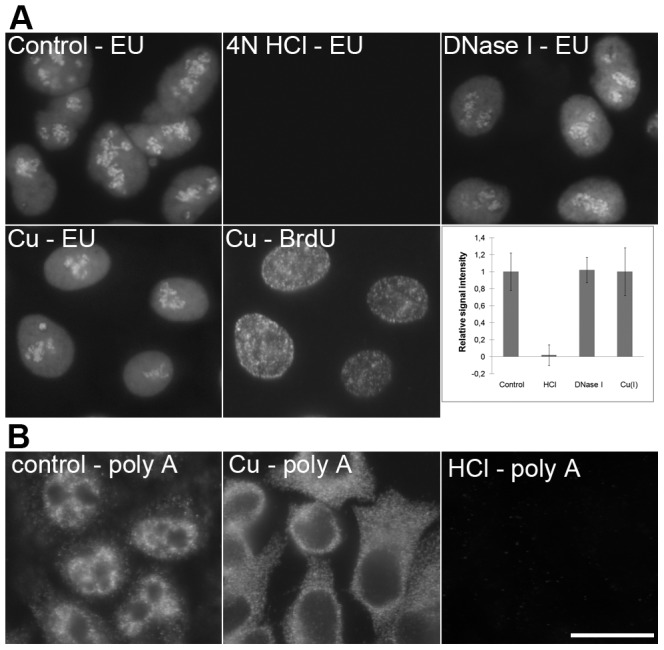
A comparison of the methods based on HCl, DNase I and copper(I) ions. **A**) The detection of the EU signal in non-treated cells (control; the cells were just fixed and permeabilized without any additional treatment), cells treated with 4N HCl, DNase I or with a standard two-step procedure (Cu – EU) is shown. All of the images have been acquired at 17 ms. A simultaneous detection of the BrdU-labeled newly replicated DNA in copper-treated cells from the Cu – EU image is shown in the image labeled as Cu – BrdU (the acquisition time was 230 ms). The graph shows the relative signal intensities of particular kinds of the treatments used. Bars: 20 µm. **B**) The detection of polyadenylated RNA in non-treated cells (control; the cells were just fixed and permeabilized without any additional treatment), in cells treated with a standard two-step procedure (Cu) and in cells treated with 4 N HCl (HCl) is shown. Polyadenylated RNA was detected according to [29]. Bar: 20 µm.

On the other hand, copper(I) treatment resulted in a progressive loss of the nucleolar signal of fibrillarin and RNA polymerase I (not shown). Although this seems to be attributable to the cleavage of the major component of nucleoli – ribosomal RNA, our results with the detection of the transcription signal by means of EU in the copper(I)-treated cells (Figure 8A) did not show any decrease of the transcription signal. In this respect, the acid treatment resulted in the complete loss of the EU signal (Figure 8A).

More light on the possible mechanism of the loss of the fibrillarin and RNA polymerase I signal was shed by the results of the detection of polyadenylated RNA (Figure 8B). We observed a complete loss of the nuclear signal and the significant increase of the cytoplasmic signal after the copper(I) treatment. In contrast, the acid treatment resulted in the complete loss of the signal in both the nucleus and the cytoplasm. It argues for a mechanism based rather on the detachment of the RNA molecules followed by the release of RNA polymerase I or fibrillarin than on massive RNA cleavage.

**Figure 9:**
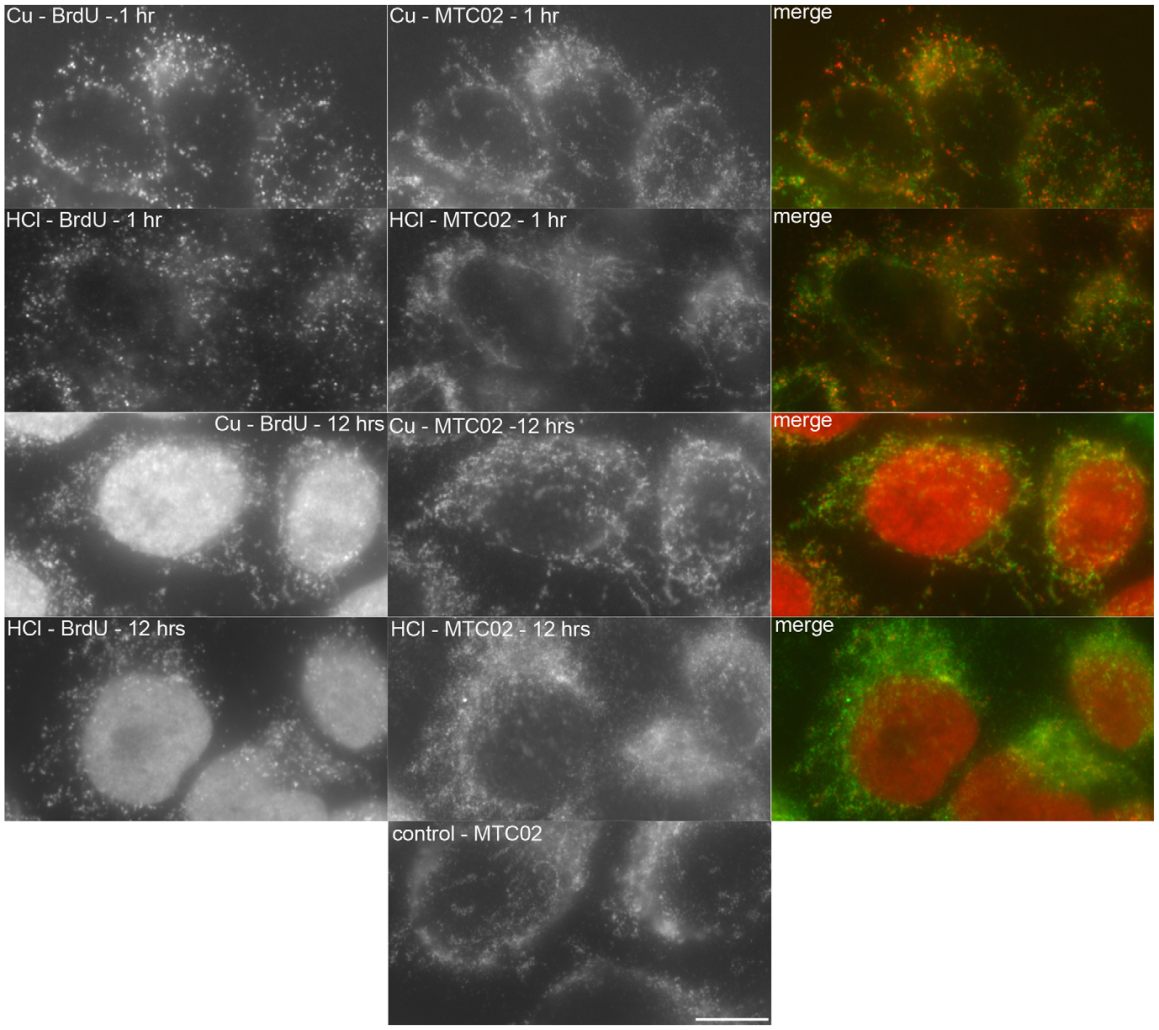
The detection of the BrdU-labeled DNA in mitochondria. The detection of the BrdU-labeled DNA (red in the color image) after a 1- and 12-hour BrdU pulse in cells treated with 0.5 M HCl for 20 minutes (the acquisition times were 335 ms and 33 ms for the 1- and 12-hour labeling, respectively) or with 4 mM copper(I) for 60 seconds followed by exonuclease III cleavage (the acquisition times were 54 ms and 6 ms for the 1- and 12-hour labeling, respectively) is shown. A simultaneous colocalization with the mitochondrial marker MTC02 has been performed for both methods (green in the color image). In this case, the acquisition times were: 260 ms for the copper(I) cleavage and 960 ms for the HCl treatment. The localization of the mitochondrial marker MTC02 in the copper(I) or HCl non-treated cells is shown in the image labeled as control (the acquisition time was 200 ms). Bar: 10 µm.

In the next series of experiments, we tested the sensitivity of the copper(I)-based method. From the results obtained, it is evident that this method is sensitive enough to visualize the mitochondrial genome replication even after a 1-hour incubation with BrdU (Figure 9). The only prerequisite for the effective resolution of the replication of mitochondrial genomes by wide-field microscopy is lowering of the nuclear signal that otherwise masks the mitochondrial signal. It was reached by shortening the incubation time in copper(I) solution. Sixty seconds sufficed for the effective visualization of the mitochondrial genome replication and the partial suppression of the nuclear signal. Also in this case, the copper(I) method provided a higher BrdU-derived signal than 0.5 N HCl. Commonly, much longer acquisition times were necessary in the case of the HCl treatment to obtain the signal intensity provided by the copper(I) treatment (see the legend to Figure 9).

## Discussion

We have described a new method of tracking the BrdU-labeled DNA based on copper(I)-mediated DNA cleavage. The principle of oxidative-radical reaction causing DNA cleavage has been described earlier by several authors [reviewed in 15], but it has never been employed to detect the replicational signal in cells. Although the cleavage solution typically contains also hydrogen peroxide (e.g. [16], [17], [19]), our experiments with the addition of hydrogen peroxide showed its very destructive effect on the cellular proteins. Our results have shown that in the presence of oxygen in the cleavage solution there is no requirement for the addition of hydrogen peroxide.

The results with the cleavage of plasmid DNA by copper(I) ions have shown that the average size of the cleaved DNA strands is around 300 bps. It is in the agreement with authors [28] who observed that the DNA fragments size is between 100–300 bps. Although the situation in fixed cells is more difficult to study and a higher concentration of copper(I) ions is required, the intensive signal after the detection of the DNA breaks by means of DNA polymerase I showed that these breaks are so close to one another that they can be used for the detection of DNA replication signal.

According to our results, the radical oxidative attacks also happen on nucleobases, but when compared to the attack on the deoxyribose moiety they exhibit much less frequent damage of DNA. It is also obvious that at least two forms of oxygen radicals are employed during the cleavage. According to our data, the important role is played by singlet oxygen and probably also by the superoxide radical. Contrary to previous studies (e.g. [15], [16]), our results did not confirm the role of hydroxyl radicals in this cleavage reaction. However, in this respect, there is no unity about the role and formation of hydroxyl radicals as also other studies have indicated that hydroxyl radicals do not cause DNA cleavage [29]–[31]. Moreover, we have shown that there are phosphate groups at the 3′ ends of these gaps.

According to our results, the described method of the detection of replicated DNA is very useful alternative to the presently-used approaches using mineral acids, hydroxides or strictly enzymatic approaches [1]–[5]. The main advantage of our approach is the minimization of protein degradation as compared to the use of mineral acids or hydroxides and a much higher signal to noise ratio in the case of BrdU localization than provided by the strictly enzymatic methods in the case of the BrdU-labeled DNA localization. Surprisingly, copper(I) treatment resulted even in a high increase of the labeling of the replicational clamp – PCNA – and the replication factor Cdc45 as compared to the non-treated cells. Importantly, the two alternative methods based on the acid treatment and DNase I resulted in the progressive lowering or loss of the specific signals. This lowering can be attributed in both cases rather to DNA cleavage than to protein degradation, because in the case of DNA non-bound proteins such as the SC35 protein or coilin, no such strong effect was observed after the acid treatment.

We have also shown that the Cu(I) treatment leads to a progressive loss of the signal provided by two nucleolar components, fibrillarin and RNA polymerase I. Although this question was not addressed more specifically, it seems that it resulted rather from the detachment of the mildly-fixed protein components than from the denaturation or effective scission of RNA, which is the major component of the nucleolar “scaffold”. In this respect, we have shown that the described method enables colocalization experiments with the newly-synthesized RNA. These data can also explain the progressive increase of the signal provided by anti-PCNA and anti-Cdc-45 antibodies after the copper(I) treatment, as the target proteins can be masked by extractable components that are washed away during the copper(I) treatment.

According to our results, the method described is especially convenient in the case of a simultaneous detection of the BrdU-labeled nuclear or mitochondrial DNA with most proteins and/or some RNA molecules. The examples include the simultaneous labeling of DNA replication and chromatin-bound proteins and/or transcripts labeled by EU. The method described can also be useful when the simultaneous detection of the BrdU-labeled DNA and overall DNA stained by DNA dyes is to be performed.

## References

[1] Ageno M, Dore E, Frontali C (1969) The alkaline denaturation of DNA. Biophys J 9: 1281–1311. 4982056 10.1016/S0006-3495(69)86452-0 PMC1367631

[2] Dimitrova DS, Berezney R (2002) The spatio-temporal organization of DNA replication sites is identical in primary, immortalized and transformed mammalian cells. J Cell Sci 115: 4037–4051. 12356909 10.1242/jcs.00087

[3] Jackson DA, Pombo A (1998) Replicon clusters are stable units of chromosome structure: evidence that nuclear organization contributes to the efficient activation and propagation of S phase in human cells. J Cell Biol 140: 1285–1295. 9508763 10.1083/jcb.140.6.1285 PMC2132671

[4] Kennedy BK, Barbie DA, Classon M, Dyson N, Harlow E (2000) Nuclear organization of DNA replication in primary mammalian cells. Genes Dev 14: 2855–2868. 11090133 10.1101/gad.842600 PMC317063

[5] Stanojcic S, Lemaitre JM, Brodolin K, Danis E, Mechali M (2008) In Xenopus egg extracts, DNA replication initiates preferentially at or near asymmetric AT sequences. Mol Cell Biol 28: 5265–5274. 18573882 10.1128/MCB.00181-08 PMC2519731

[6] Tkatchenko AV (2006) Whole-mount BrdU staining of proliferating cells by DNase treatment: application to postnatal mammalian retina. Biotechniques 40: 29–30, 32. 16454036 10.2144/000112094

[7] Salic A, Mitchison TJ (2008) A chemical method for fast and sensitive detection of DNA synthesis in vivo. Proc Natl Acad Sci U S A 105: 2415–2420. 18272492 10.1073/pnas.0712168105 PMC2268151

[8] Diermeier-Daucher S, Clarke ST, Hill D, Vollmann-Zwerenz A, Bradford JA, et al (2009) Cell type specific applicability of 5-ethynyl-2′-deoxyuridine (EdU) for dynamic proliferation assessment in flow cytometry. Cytometry A 75: 535–546. 19235202 10.1002/cyto.a.20712

[9] Meneni S, Ott I, Sergeant CD, Sniady A, Gust R, et al (2007) 5-Alkynyl-2′-deoxyuridines: chromatography-free synthesis and cytotoxicity evaluation against human breast cancer cells. Bioorg Med Chem 15: 3082–3088. 17336074 10.1016/j.bmc.2007.01.048 PMC2577600

[10] Davis AF, Clayton DA (1996) In situ localization of mitochondrial DNA replication in intact mammalian cells. J Cell Biol 135: 883–893. 8922374 10.1083/jcb.135.4.883 PMC2133381

[11] Haines KM, Feldman EL, Lentz SI Visualization of mitochondrial DNA replication in individual cells by EdU signal amplification. J Vis Exp 10.3791/2147 PMC3159597 21113116

[12] Iborra FJ, Kimura H, Cook PR (2004) The functional organization of mitochondrial genomes in human cells. BMC Biol 2: 9. 15157274 10.1186/1741-7007-2-9 PMC425603

[13] Koberna K, Stanek D, Malinsky J, Eltsov M, Pliss A, et al (1999) Nuclear organization studied with the help of a hypotonic shift: its use permits hydrophilic molecules to enter into living cells. Chromosoma 108: 325–335. 10525969 10.1007/s004120050384

[14] Ligasova A, Raska I, Koberna K (2009) Organization of human replicon: singles or zipping couples? J Struct Biol 165: 204–213. 19063972 10.1016/j.jsb.2008.11.004 PMC2670984

[15] Pogozelski WK, Tullius TD (1998) Oxidative Strand Scission of Nucleic Acids: Routes Initiated by Hydrogen Abstraction from the Sugar Moiety. Chem Rev 98: 1089–1108. 11848926 10.1021/cr960437i

[16] Drouin R, Rodriguez H, Gao SW, Gebreyes Z, O'Connor TR, et al (1996) Cupric ion/ascorbate/hydrogen peroxide-induced DNA damage: DNA-bound copper ion primarily induces base modifications. Free Radic Biol Med 21: 261–273. 8855437 10.1016/0891-5849(96)00037-8

[17] Lloyd DR, Phillips DH (1999) Oxidative DNA damage mediated by copper(II), iron(II) and nickel(II) fenton reactions: evidence for site-specific mechanisms in the formation of double-strand breaks, 8-hydroxydeoxyguanosine and putative intrastrand cross-links. Mutat Res 424: 23–36. 10064847 10.1016/s0027-5107(99)00005-6

[18] Reed CJ, Douglas KT (1989) Single-strand cleavage of DNA by Cu(II) and thiols: a powerful chemical DNA-cleaving system. Biochem Biophys Res Commun 162: 1111–1117. 2504154 10.1016/0006-291x(89)90788-2

[19] Stoewe R, Prutz WA (1987) Copper-catalyzed DNA damage by ascorbate and hydrogen peroxide: kinetics and yield. Free Radic Biol Med 3: 97–105. 2822548 10.1016/s0891-5849(87)80003-5

[20] Johnson GRA, Nazhat NB, Saadallanazhat RA (1988) Reaction of the Aquacopper(I) Ion with Hydrogen-Peroxide - Evidence for a Cuiii (Cupryl) Intermediate. J Chem Soc, Faraday Trans 1 84: 501–510.

[21] Masarwa M, Cohen H, Meyerstein D, Hickman DL, Bakac A, et al (1988) Reactions of Low-Valent Transition-Metal Complexes with Hydrogen-Peroxide - Are They Fenton-Like or Not .1. The Case of Cu+Aq and Cr-2+Aq. J Am Chem Soc 110: 4293–4297.

[22] Sagripanti JL, Kraemer KH (1989) Site-specific oxidative DNA damage at polyguanosines produced by copper plus hydrogen peroxide. J Biol Chem 264: 1729–1734. 2912981

[23] Yamamoto K, Kawanishi S (1989) Hydroxyl free radical is not the main active species in site-specific DNA damage induced by copper (II) ion and hydrogen peroxide. J Biol Chem 264: 15435–15440. 2549063

[24] Aruoma OI, Halliwell B, Gajewski E, Dizdaroglu M (1991) Copper-ion-dependent damage to the bases in DNA in the presence of hydrogen peroxide. Biochem J 273 ((Pt 3)) 601–604. 1899997 10.1042/bj2730601 PMC1149805

[25] Burrows CJ, Muller JG (1998) Oxidative Nucleobase Modifications Leading to Strand Scission. Chem Rev 98: 1109–1152. 11848927 10.1021/cr960421s

[26] Sporbert A, Gahl A, Ankerhold R, Leonhardt H, Cardoso MC (2002) DNA polymerase clamp shows little turnover at established replication sites but sequential de novo assembly at adjacent origin clusters. Mol Cell 10: 1355–1365. 12504011 10.1016/s1097-2765(02)00729-3

[27] Ligasová A, Koberna K (2010) In situ reverse transcription: the magic of strength and anonymity. Nucleic Acids Res 38: e167. 20627869 10.1093/nar/gkq619 PMC2938209

[28] Sirbu BM, Couch FB, Feigerle JT, Bhaskara S, Hiebert SW, et al (2011) Analysis of protein dynamics at active, stalled, and collapsed replication forks. Genes Dev 25: 1320–1327. 21685366 10.1101/gad.2053211 PMC3127432

[29] Rush JD, Koppenol WH (1988) Reactions of FeIInta and FeIIedda with hydrogen peroxide. J Am Chem Soc 110: 4957–4963.

[30] Sawyer DT, Kang C, Llobet A, Redman C (1993) Fenton Reagents (1∶1 FeIILx/HOOH) React via [LxFeIIOOH(BH+)] (1) as Hydroxylases (Rh→ROH), not as Generators of Free Hydroxyl Radicals (HO). J Am Chem Soc 115: 5817–5818.

[31] Yamazaki I, Piette LH (1991) EPR spin-trapping study on the oxidizing species fomed in reaction of the ferrous ion with hydrogen peroxide. J Am Chem Soc 113: 7588–7593.

